# Assessment of metabolomic variations among individuals returning to plain areas after exposure to high altitudes: a metabolomic analysis of human plasma samples with high-altitude de-acclimatization syndrome

**DOI:** 10.3389/fmolb.2024.1375360

**Published:** 2024-06-19

**Authors:** Zhen Tan, Pan Shen, Yi Wen, Hong-yu Sun, Hong-yin Liang, Hua-ji Qie, Rui-wu Dai, Yue Gao, Zhu Huang, Wei Zhou, Li-jun Tang

**Affiliations:** ^1^ Department of General Surgery, General Hospital of Western Theater Command, Chengdu, Sichuan, China; ^2^ Pancreatic Injury and Repair Key Laboratory of Sichuan Province, General Hospital of Western Theater Command, Chengdu, Sichuan, China; ^3^ Department of Pharmaceutical Sciences, Beijing Institute of Radiation Medicine, Beijing, China; ^4^ Department of Central Lab, General Hospital of Western Theater Command, Chengdu, Sichuan, China

**Keywords:** high altitude de-acclimatization, metabolomics, metabolites, lipid oxidation, lipid metabolism

## Abstract

**Background:**

High altitude de-acclimatization (HADA) is gradually becoming a public health concern as millions of individuals of different occupations migrate to high-altitude areas for work due to economic growth in plateau areas. HADA affects people who return to lower elevations after exposure to high altitudes. It causes significant physiological and functional changes that can negatively impact health and even endanger life. However, uncertainties persist about the detailed mechanisms underlying HADA.

**Methods:**

We established a population cohort of individuals with HADA and assessed variations in metabolite composition. Plasm samples of four groups, including subjects staying at plain (P) and high altitude (H) as well as subjects suffering from HADA syndrome with almost no reaction (r3) and mild-to-moderate reaction (R3) after returning to plain from high altitude, were collected and analyzed by Liquid Chromatography-Mass Spectrometry metabolomic. Multivariate statistical analyses were used to explore significant differences and potential clinical prospect of metabolites.

**Result:**

Although significantly different on current HADAS diagnostic symptom score, there were no differences in 17 usual clinical indices between r3 and R3. Further multivariate analyses showed isolated clustering distribution of the metabolites among the four groups, suggesting significant differences in their metabolic characteristics. Through K-means clustering analysis, we identified 235 metabolites that exhibited patterns of abundance change consistent with phenotype of HADA syndrome. Pathway enrichment analysis indicated a high influence of polyunsaturated fatty acids under high-altitude conditions. We compared the metabolites between R3 and r3 and found 107 metabolites with differential abundance involved in lipid metabolism and oxidation, suggesting their potential role in the regulation of oxidative stress homeostasis. Among them, four metabolites might play a key role in the occurrence of HADA, including 11-beta-hydroxyandrosterone-3-glucuronide, 5-methoxyindoleacetate, 9,10-epoxyoctadecenoic acid, and PysoPC (20:5).

**Conclusion:**

We observed the dynamic variation in the metabolic process of HADA. Levels of four metabolites, which might be provoking HADA mediated through lipid metabolism and oxidation, were expected to be explore prospective indices for HADA. Additionally, metabolomics was more efficient in identifying environmental risk factors than clinical examination when dramatic metabolic disturbances underlying the difference in symptoms were detected, providing new insights into the molecular mechanisms of HADAS.

## 1 Introduction

High altitude de-acclimatization (HADA) is a serious and complex process resulting from the loss of high-altitude acclimatization, provoking physiological and functional changes in individuals who have adapted to a high altitude before returning to a lower elevation or plain area (He et al., 2013; Paul et al., 2023). These changes in physiological functions were first reported in 1908 among high-altitude explorers returning to sea level (Ward, 1908). Millions of individuals, including explorers, athletes, military personnels, and workers, temporarily migrated to high-altitude areas for work due to the recent economic growth in plateau areas of China (He et al., 2013). However, most of them experienced HADA syndrome (HADAS) upon returning to lower altitudes each year. Similar to the results of our previous works, Cui et al. reported that 71% of individuals living at high altitudes for over 10 years presented a series of symptoms of HADAS (Cui et al., 1998), and our previous study (ZHOU et al., 2020) showed that the overall detection rate of HADAS was 76%. The most common clinical symptoms were neurological symptoms, followed by respiratory, circulatory, and digestive symptoms. Furthermore, another study generalized that the symptoms of HADAS included insomnia, sleepiness, memory loss, unresponsiveness, headache, fidgetiness, coughing, throat pain or discomfort, chest tightness, sputum, becoming flustered, decreased appetite, increased appetite, diarrhea, abdominal pain, abdominal distention, arthralgia, and lumbago, which notably lowered the quality of life (Zhou SY et al., 2012; ZHOU et al., 2020). Worse still, these clinical symptoms could persist for many years and even force 1‰ of severe cases to return to high altitudes (Zhang et al., 2012).

Although HADA has gradually become a public health issue and received more attention, limited understanding of HADA make its diagnostic criteria and treatment options still less precise. To our knowledge, the diagnostic criteria of HADAS constitute essential and auxiliary criteria as well as symptom scores of HADAS (Zhou SY et al., 2012; He et al., 2013), which is a complex and multiple-symptom system mainly based on subjective feelings, making the evaluation of HADAS a heavy workload for physicians and researchers. Therefore, improve the understanding for exploring prospective indices for HADAS based on existing criteria of symptom scores is necessary. In many ways, researchers have primarily focused on the mechanisms of HADA to develop biomarkers. Previous studies showed that HADAS affects individuals living in hypoxic conditions before quickly returning to normoxic environments and is associated with rapidly increased levels of PaO_2_ and SO_2_ (Ozaki et al., 2003; Sureda et al., 2004). Hypoxia/reoxygenation (H/R) resulting from environmental changes, which induced oxidative stress, the production of reactive oxygen species (ROS), and increased generation of proinflammatory mediators, was considered the main factor promoting HADAS (Yang et al., 2023). However, the detailed mechanisms underlying these changes in high- and low-altitude environments remained unclear.

Metabolomics has regarded as a powerful tool to help us to improve the understanding of the field of high altitude acclimatization relying on analysis platforms to evaluate metabolic variations induced by environmental stimulation (Nicholson et al., 1999; O'Brien et al., 2019). Gao et al. conducted a longitudinal cohort study (Gao et al., 2023) using a non-targeted liquid chromatography-mass spectrometry (LC-MS) metabolomics analysis and showed that 13 differential metabolites were significantly modified after exposure to high-altitude hypoxia, and six of the metabolites were still affected when returning to a low-altitude. However, there are limited cross-sectional studies on the metabolic changes in individuals who suffered from HADAS after returning to low elevations from a high-altitude plateau. The present cross-sectional study used non-targeted LC-MS metabolomics analysis based on the existing criteria of symptom scores to investigate the metabolic differences in the human plasma samples with HADAS after exposure to high altitude (3,600 m) and return to a plain area (500 m).

## 2 Materials and methods

### 2.1 Blood samples of subjects and ethical approvals

All the subjects (32 healthy males, 26.2 ± 4.1 years old) were selected randomly and divided into four groups, including a high altitude (H, *n* = 8) group, almost no reaction HADAS (r3, *n* = 8) group, moderate reaction HADAS (R3, *n* = 8) group, and plain (P, *n* = 8) group. The subjects of H, R3, and r3 groups from Chengdu (500 m) had worked in Pi-Shan County of Xin-Jiang district (3,600 m) for 1 year and then returned to the Chengdu plain. Subjects of R3 and r3 groups, whose morning fasting venous blood samples were collected before being evaluated 3 days after they returned, were diagnosed with HADAS according to existing diagnostic and scoring criteria. Morning fasting venous blood samples of the H group subjects were collected at the plateau before they returned to the plain and used as the high-altitude control group. In addition, subjects of the P group who had always lived in Chengdu were regarded as the plain control group. All the blood samples (3 mL) of the above four group subjects were centrifuged at 4,000 rpm for 10 min to separate serum plasma and stored at −80 °C before assay. Clinical characteristics including age, body mass index (BMI), heart rate (HR), oxygen saturation (SaO_2_), red blood cell count (RBC), hemoglobin (HGB), hematocrit (HCT), blood urea nitrogen (BUN), creatinine (CREA), uric acid, total protein (TP), albumin (ALB), globulin (GLB), glutamic oxaloacetic transaminase (AST), glutamic pyruvic transaminase (ALT), total bilirubin, and indirect bilirubin (IBIL) were measured (Supplementary Table S1). All subjects provided written informed consent. The Medical Ethical Committee of the General Hospital of Western Theater Command PLA approved the present study.

### 2.2 Diagnostic scoring criteria for HADAS

According to the present essential diagnostic criteria, individuals under 60 years old who recently returned to a lower altitude from a higher altitude and present with of three or more relevant symptoms that persist after 3 days of simple medication are said to have HADAS. These symptoms included sleepiness, fatigue, unresponsiveness, insomnia, fidgety, memory loss, throat pain or discomfort, headache, expectoration, coughing, flustering, chest tightness, decreased appetite, increased appetite, diarrhea, expectoration, abdominal pain, abdominal distention, arthralgia or lumbago.

The scoring criteria of each symptom were as follows: 0, mild symptoms with no impact on daily life; 1, Mild symptoms with a slight impact on daily life, ameliorated after drug regimen; 2, severe symptoms that affect daily life but somewhat alleviated after drug regimen; 3, severe symptoms that affect daily life, with no significant relief after drug regimen. The grading of HADAS was based on the total symptom points, which is the sum of all the points assigned to each symptom. Total scores of 0–5, 6–15, 16–25, and ≥26 indicated almost no reaction (±), mild reaction (+), moderate reaction (++), and severe reaction (+++), respectively. In the present study, the total point of the R3 group was 15.625 ± 2.973 and that of the r3 group was 2.625 ± 1.060.

The exclusion criteria included symptoms directly attributable to primary diseases affecting the cardiovascular, hematological, respiratory, urinary, and nervous systems, cancer or leukemia, and a recent history of upper respiratory tract infection, influenza, or symptoms caused by infectious agents.

### 2.3 Non-targeted liquid chromatography-mass spectrometry (UHPLC-MS/MS) metabolomic analysis

Before UHPLC-MS/MS metabolomic analysis were taken, samples were prepared by mixing 200 μL of plasma with 300 μL methanol-analytical grade water (V: V = 1:4) solution using a vortex for 30 s to precipitate protein, and then centrifuged (13,000 rpm 4°C, 10 min). Quality control (QC) samples were generated by mixing equal volume samples from each test sample and were injected between each group. QC samples were used to assess instrument stability and data quality throughout the study by the procedure including QC sample screening by relative standard deviation, principal component analysis (PCA) of QC samples, evaluation of QC metabolites intensity distribution by Boxplot, and stability evaluation of QC samples by Hierarchical clustering. A Dionex U3000 UHPLC system (Thermo Fisher, MA, United States) and ACQUITY UPLC HSS T3 column (100 × 2.1 mm, 1.8 μm) (Waters, MA, United States) with a 16 min linear gradient at a flow rate of 0.35 mL/min was used for LC. Eluent A (0.1% formic acid in water) and eluent B (0.1% methanol) were used for the mobile phase. The injected sample volume was 2 μL, and the column oven was maintained at 45 °C. LC was coupled with a Orbitrap Q Exactive (QE) plus a high-resolution mass spectrometer (Thermo Fisher). The QE plus high-resolution mass spectrometer was operated in both positive and negative polarity modes with spray voltage of 3800 V and −3000V respectively, a sheath gas flow rate of 35 arb, an aux gas flow rate of eight arb, capillary temperature of 320 °C, aux gas heater temperature of 350°C. Full scan mass spectrometry data were acquired in a mass range of 100–1,200 m/z.

### 2.4 Data processing and statistical analyses

The raw data files generated by non-targeted LC-MS/MS metabolomic analysis were processed using Progenesis QI (v2.3) to perform qualitative and quantitative analysis as well as standardized pretreatment for each metabolite, including baseline filtering, integration, peak identification, peak alignment, and normalization. Peaks with a signal/noise ratio (SNR) over 50 were normalized to the intensity of the total spectral intensity. Based on fragment ions, molecular ion peaks and addictive ions, the normalized data mentioned above was applied to predict the molecular formula. Metabolite identifications, which meet three criteria including narrow window retention index, accurate mass with variation less than 10 ppm and MS/MS spectra with high forward and reverse scores based on comparisons of ions presented in the experimental spectrum to that in the library spectrum entries, based on the accurate mass number, secondary fragments, and isotope distribution were established by matching against the online spectral libraries to obtain the accurate qualitative and relative quantitative results. Most isomers could be distinguished by these criteria and the identified metabolites meet the level 1 requirements by the Metabolomics Standards Initiative, and a few metabolites, whose MS/MS spectra were computationally matched, fulfill the criteria for levels 2-4. Metabolites were initially annotated using The Human Metabolome Database (HMDB, v5.0), Lipidmaps (v2.3), and METLIN. To exclude metabolites such as drug-derived exogenous compounds that may not naturally occur in humans, all metabolites were further retained if they were documented in both HMDB and Lipidmaps, followed by additional screening and curation through artificial means. PCA, partial least squares discriminant analysis (PLS-DA), and orthogonal partial least squares analysis (OPLS-DA) were performed to analyze the overall differences in metabolic profiles using the web tool Metaboanalyst (v5.0) (Pang et al., 2021). The Student’s t-test and fold change between groups were further performed to calculate the statistical significance. The metabolites with variable importance in projection value calculated by OPLS-DA > 1 and *p*-value <0.05 adjusted by the Benjaminiand Hochberg method, were considered to be metabolites with differential abundance. Volcano plots and heat maps were used to filter and screen differential metabolites of interest. Kyoto encyclopedia of genes and genomes (KEGG) pathway enrichment analysis were performed using Metaboanalyst to measure the close correlation between metabolites and explore the potential mechanism of metabolic pathway changes. The statistical analysis involved conducting one-way analysis of variance and Wilcoxon rank sum tests to assess the differences in clinical indicators and metabolites between the groups. Receiver operating characteristic (ROC) curve for each metabolite was generated using the R package pROC (v1.18.4), and the area under the curve (AUC) value for each ROC curve was obtained.

## 3 Results

### 3.1 Clinical characteristics of subjects after exposure to a high altitude and returning to a plain area

**FIGURE 1:**
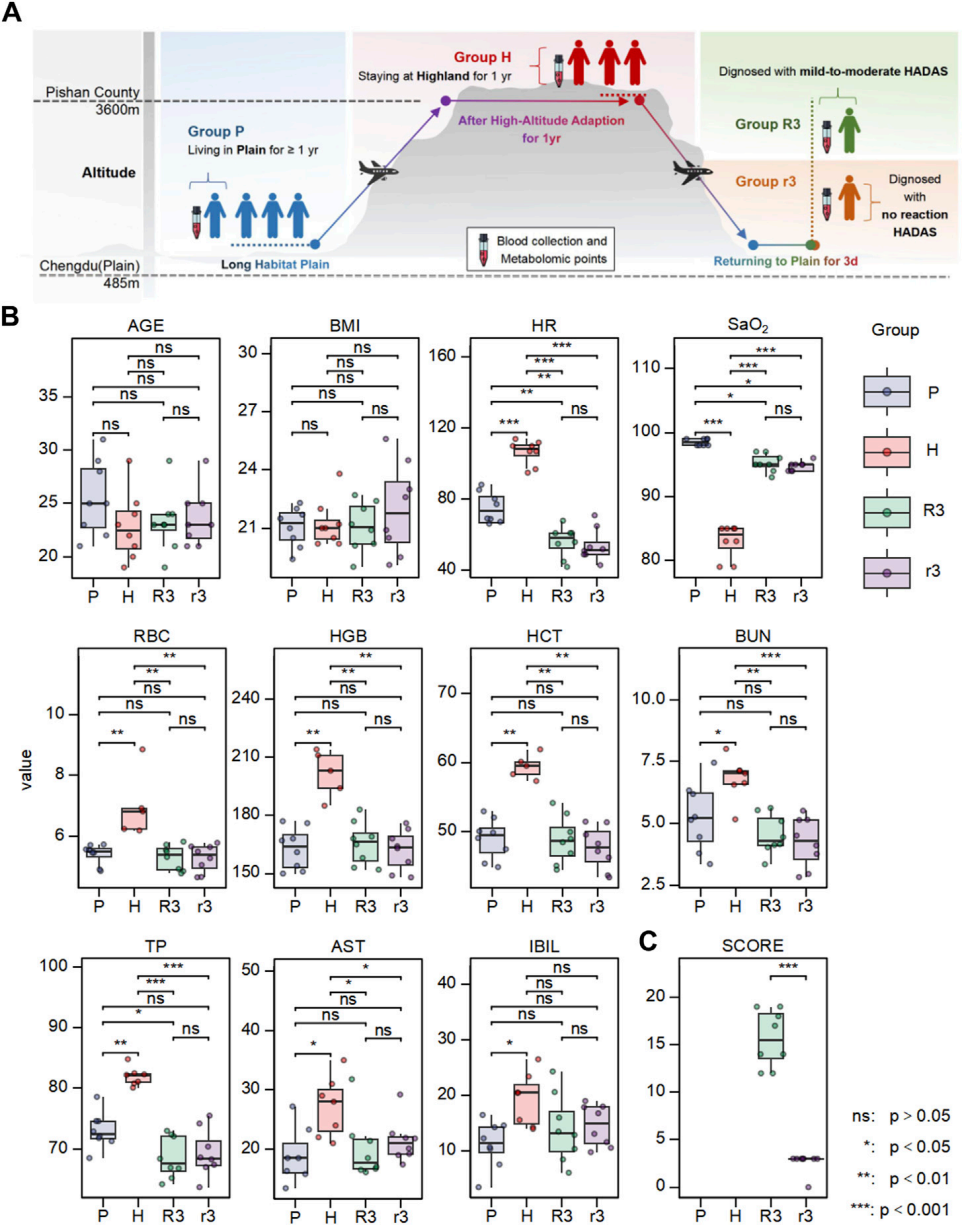
The experimental design and the variation of clinical phenotype in different group. **(A)** The overall design of this work. **(B)** The differences of clinical characteristics including age, BMI, HR, SaO_2_, RBC, HBG, HCT, UA, TP, AST, IBIL. **(C)** The difference of HADAS symptom scores between r3 and R3 group. Ns, *p* > 0.05; *, *p* < 0.05; **, *p* < 0.01; ***, *p* < 0.001.

The overall study design is shown in Figure 1A. We split the study participants into four groups: the plain group (living in the plain and not going to the highland for at least 1 year, P), high altitude group (staying at the highland for 1 year, H), returned group with no reaction HADAS (staying in the highland for 1 year before returning to the plain for 3 days, suffering from no reaction HADAS, r3), and returned group with mild-to-moderate HADAS (staying at the highland for 1 year before returning to the plain for 3 days, suffering from mild-to-moderate HADAS, R3). The plasma samples of each group were collected and related clinical parameters were tested. The clinical characteristics of the four groups are summarized in Figure 1B and Supplementary Table S1. The value of parameters including HR, RBC, HGB, HCT, BUN, TP, AST, and IBIL in H group were significantly higher than in P group, and the value of SaO_2_ in H group was lower than in P group. These values of parameters presented significantly reciprocal change in r3 and R3 group (*p* < 0.05). These change trends were consistent with previous studies, suggesting that the changes in these clinical characteristics caused by exposure to high altitude would mostly normalize after returning to the plain for 3 days. Notably, there were no differences in all these characteristics (*p* > 0.05) between the r3 and R3 groups, although there was a significant difference in the total symptom score points (*p* < 0.0001) (Figure 1C). These findings suggest commonly used clinical examination indices were not specific in diagnosing HADAS and highlight the need to explore more efficient and warning indices.

### 3.2 Metabolomic profiling in human plasma of subjects in different environments

**FIGURE 2:**
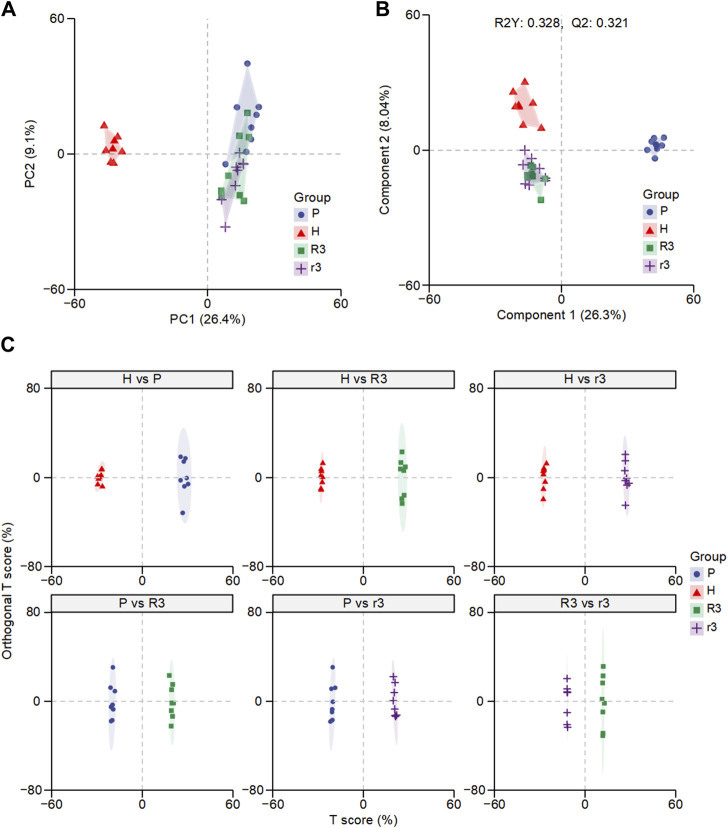
The overall difference of plasma metabolome in different groups. **(A)** Principal component analysis (PCA) of the H, P, R3 and r3 groups. **(B)** Partial least squares-discriminant analysis (PLS-DA) of the H, P, R3 and r3 groups. **(C)** Orthogonal PLS-DA (OPLS-DA) of each comparison.

Non-targeted LC-MS/MS was performed to detect the plasma metabolic profiling. The data acquired was initially used to establish a PCA model, which provided an overview of all the groups. The PCA score plot showed that QC samples clustered together, suggesting good QC repeatability and analysis system stability, and the metabolic profiles of the H and P groups were easy to separate (Figure 2A; Supplementary Figure S1). However, the profiles of the r3 and R3 groups could not be distinguished from the P group, suggesting that the characteristics of r3 and R3 resembled that of the P group, probably because the samples were all collected in the low-altitude environment. PLS-DA model, a supervised pattern recognition method, explained 32.8% of the original data with acceptable prediction ability (Q_2_ = 0.321) and revealed a more prominent trend of metabolic change, where points for the P group were separated from those for the r3 and R3 groups (Figure 2B). Thought-provoking points showed that the metabolic profiling of the H and r3/R3 were more similar. In contrast, the metabolic profiling of the r3 and R3 groups could not be distinguished on the second principal component (Figure 2B). To further investigate the difference in plasma metabolism between the r3 and R3 groups, OPLS-DA was performed on pairwise metabolite data, consisting of the P, H, r3, and R3 groups and employing range normalization for scaling (Figure 2C). The Q_2_ and R_2_Y of each two groups were shown in Supplementary Table S3, accompanied by a permutation test conducted on each OPLS-DA result across various comparison groups (Supplementary Figure S2). OPLS-DA results revealed significant differences between each pair of groups, identifying that the r3 and R3 groups possessed distinct metabolic characteristics, although the predictive performance of the models appeared to be limited for r3 and R3 group. These findings provide evidence that substantial metabolic changes occur in individuals affected by changes in altitude, suggesting the involvement of specific metabolic pathways as driving factors.

### 3.3 Dynamic variation tendency in the abundance of plasma metabolites associated with the change of environment

**FIGURE 3:**
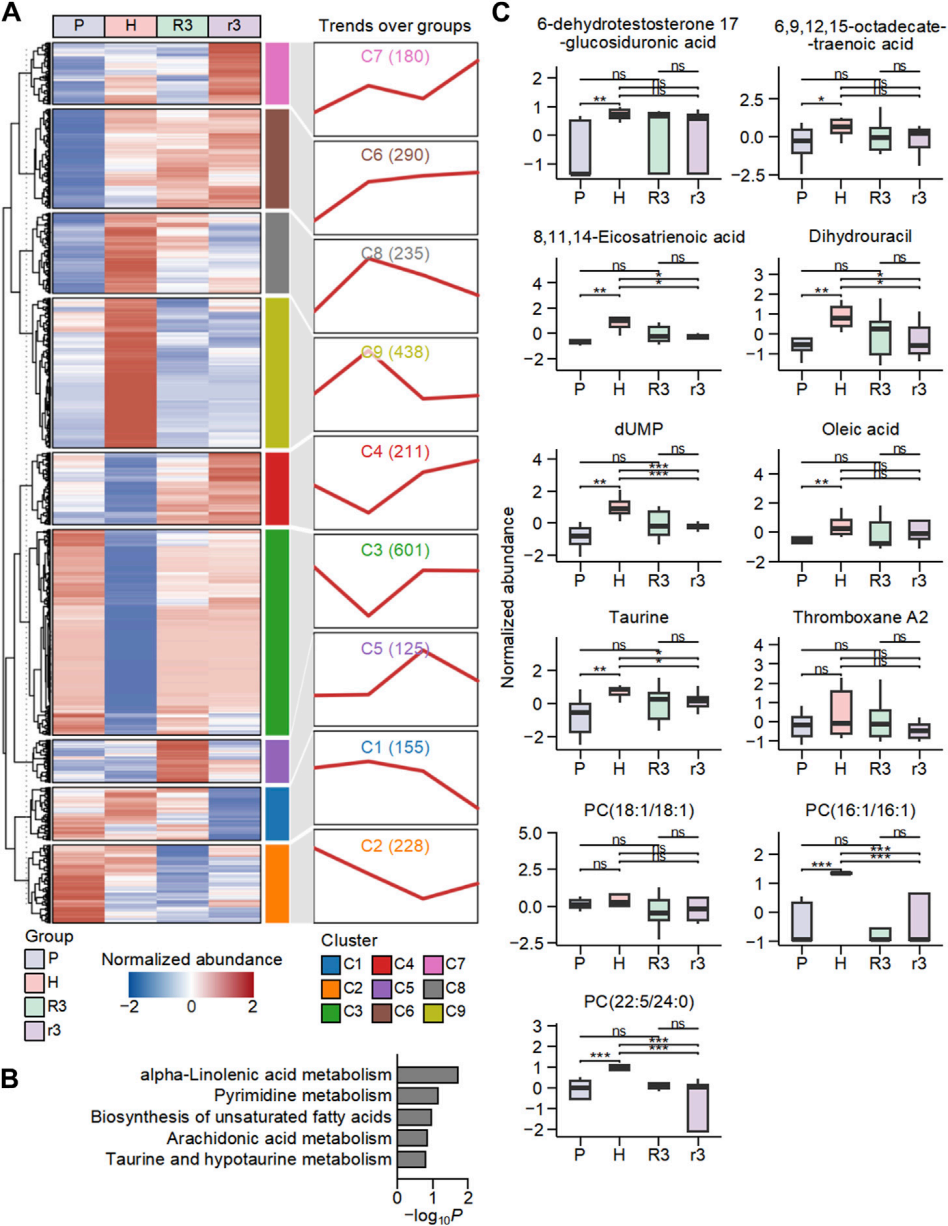
The dynamic changes in the abundance of metabolites in human plasma associated with the environment. **(A)** The classification of metabolites in P, H, R3 and r3 groups based on K-means clustering. The heat map on the left showed the mean abundance of metabolites in each group, and the line chart on the right displayed the change pattern over groups of metabolites in each cluster. Numbers in parentheses represent the number of metabolites in each cluster. **(B)** Top five enriched Kyoto encyclopedia of genes and genomes (KEGG) pathways based on metabolites in cluster 8 (C8). **(C)** The abundance changes of metabolites in the C8-enriched pathways in the four groups. Ns, *p* > 0.05; *, *p* < 0.05; **, *p* < 0.01; ***, *p* < 0.001.

A total of 2,461 metabolites were screened and obtained from plasma samples across the four groups. Initially, K-means clustering was employed to categorize all metabolites across all samples, yet yielded few insights conducive to the analysis of the HADA (Supplementary Figure S3). To delve deeper into the dynamic shifts in metabolite abundance linked to environmental factors, a subsequent K-means clustering was conducted based on trends in the mean normalized abundance of all metabolites per group, yielding eight clusters (Figure 3A, Supplementary Table S4). Of particular interest was cluster 8 (C8), which comprised 235 metabolites exhibiting lower abundances in low-altitude regions and higher abundances in high-altitude areas (Figure 3A). This observation prompted further investigation, as the trend aligns with the HADAS phenotype (Supplementary Figure S4). Upon returning from high altitude to plain areas, individuals with high symptom scores of HADAS demonstrated sustained high abundance of these metabolites, while individuals with low symptom scores showed a deficit in their abundance.

To further investigate the functions of metabolites in C8, we conducted an enrichment analysis to analyze the KEGG pathways associated with these metabolites (Figure 3B). The top five enriched KEGG pathways associated with metabolites in C8 were found to be alpha-linolenic acid metabolism, pyrimidine metabolism, biosynthesis of unsaturated fatty acids, arachidonic acid metabolism, taurine and hypotaurine metabolism. These enriched pathways indicate that the metabolism of polyunsaturated fatty acids was highly influenced by high-altitude conditions. Polyunsaturated fatty acids, such as alpha-linolenic acid, are essential components of cell membranes that are important in various physiological processes, and alterations in their metabolism could promote significant implications for lipid oxidation and oxidative stress levels in the body (Yuan et al., 2022; Bertoni et al., 2023). The involvement of pathways such as taurine and hypotaurine metabolism, arachidonic acid metabolism, and pyrimidine metabolism further supports the notion that high altitude affects the metabolism of lipids and related compounds. These pathways are closely linked to energy metabolism and lipid homeostasis (Millanvoye-Van Brussel et al., 1985; Le et al., 2013; Wang et al., 2020; Trostchansky et al., 2021; Xu et al., 2022; Kp and Martin, 2023). These results suggest that the metabolism of polyunsaturated fatty acids was significantly influenced by high altitude, potentially leading to alterations in lipid oxidation and levels of oxidative stress within the body.

Specifically, the metabolites in these KEGG pathways within C8 included 6-dehydrotestosterone 17-glucosiduronic acid, 6,9,12,15-octadecatetraenoic acid (Stearidonic acid), 8,11,14-Eicosatrienoic acid (DGLA), Dihydrouracil, dUMP, oleic acid, taurine, thromboxane A2, and three phosphatidylcholines (Figure 3C). Among them, phosphatidylcholine and thromboxane A2 were reported to be involved in the activation of lipid oxidation during high-altitude adaption (Ternovoĭ and Iakovlev, 1993; Bashir et al., 2015). Stearidonic acid, DGLA, and oleic acid have been implicated in lipid metabolism and oxidative stress (Wang et al., 2012; Whelan et al., 2012; Tie et al., 2021). These findings highlight the importance of examining the impact of high altitude on metabolic pathways involved in lipid metabolism and oxidative stress regulation, which may have implications for understanding the physiological adaptations and health consequences associated with altitude changes.

### 3.4 Metabolic dysregulation of high-altitude de-acclimatization syndrome under different total symptom scores

**FIGURE 4:**
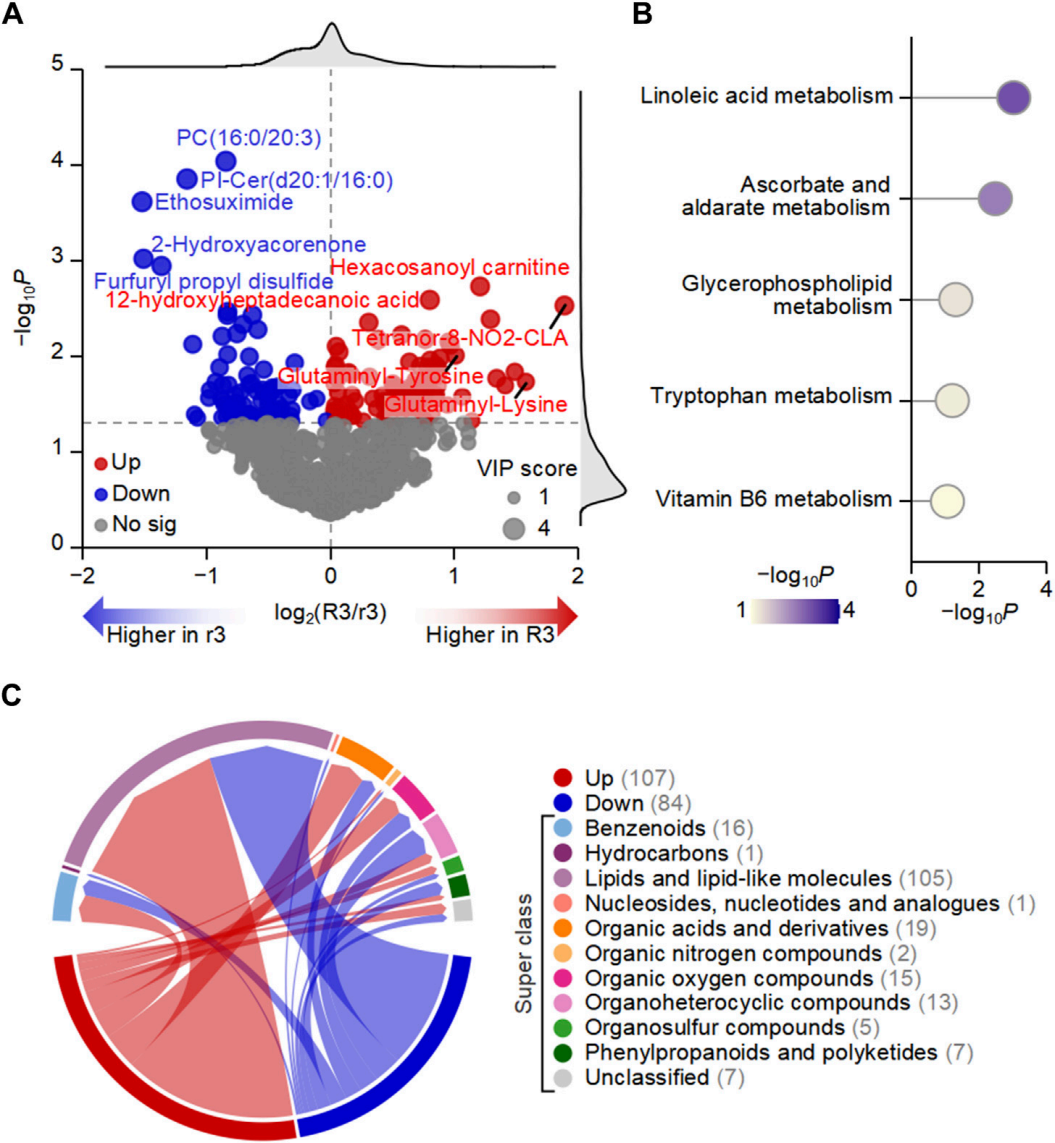
Metabolic dysregulation may be one of the important driving factors of high-altitude de-acclimatization syndrome. **(A)** Volcano plot showed that the differences in the abundance of plasma metabolites between R3 and r3 groups. **(B)** Chord diagram displaying the number of up- and downregulated metabolites in different categories. Numbers in parentheses represent the number of metabolites in each category. **(C)** Top five enriched KEGG pathways based on metabolites with differential abundance between R3 and r3 groups.

To further identify the metabolic dysregulation of HADAS under different total symptom scores, we screened differential metabolites between the R3 and r3 groups. There were 107 upregulated and 84 downregulated metabolites, as shown in the volcano plot (Figure 4A). All the up and downregulated metabolites were divided into 10 categories in the chord diagram (Figure 4B), which contained super classes with top five highest number including 105 lipids and lipid-like molecules, 19 organic acids and derivatives, 16 benzenoids, 15 organic oxygen compounds, and 13 organoheterocyclic compounds. These findings further confirmed that changes in the abundance of lipids play a significant role in driving the metabolic alterations observed in HADAS. The pathway enrichment analysis based on these metabolites revealed the top five enriched metabolic pathways, including linoleic acid metabolism, ascorbate and aldarate metabolism, glycerophospholipid metabolism, tryptophan metabolism, and vitamin B6 metabolism (Figure 4C). Among them, linoleic acid metabolism is closely linked to the metabolism of polyunsaturated fatty acids, which are susceptible to lipid oxidation and can generate ROS leading to oxidative stress (Moran et al., 2000; Yuan et al., 2022). Ascorbate and aldarate metabolism are associated with antioxidant defense mechanisms that help protect cells from oxidative damage (Yi et al., 2021). Glycerophospholipid metabolism exerted critical roles in cardiotoxicity via imbalanced mitochondrial dynamics as well that the synthesis and degradation of glycerophospholipids was one of the most highly regulated metabolisms across the 24-h cycle in terms of total lipid content and enzyme activity in the nervous system (Guido et al., 2022; Li et al., 2022). These might be associated with the symptoms of circulative and nervous system after HADAS. Tryptophan metabolism is not only essential for protein synthesis but also plays a role in the production of important metabolites such as kynurenine, which is involved in the regulation of oxidative stress and inflammation (Xue et al., 2023). Vitamin B6 metabolism is critical for the synthesis of the coenzyme pyridoxal phosphate, which participates in numerous enzymatic reactions, including those involved in amino acid and lipid metabolism (Blachier et al., 2020). Disruptions in these metabolic pathways can potentially affect lipid oxidation and oxidative stress levels, implying the potential involvement of metabolites with differential abundance in lipid oxidation and oxidative stress regulation in HADAS.

**FIGURE 5:**
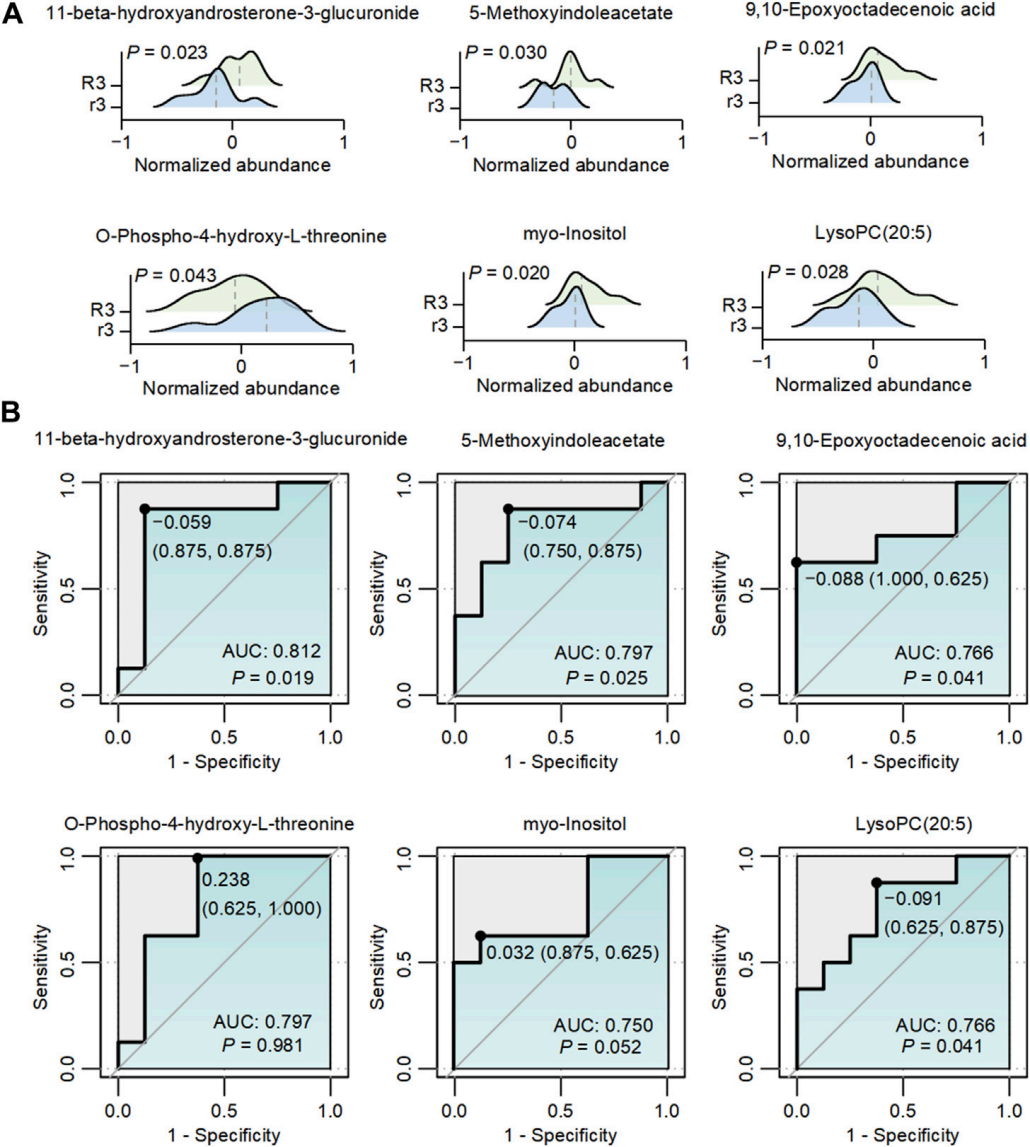
The abundance changes of metabolites with different abundances in R3 and r3 groups. **(A)** The dynamic changes in six metabolites associated with Figure 4B between the two groups. The gray dashed lines represented the median abundance in each group. **(B)** ROC graphs of six metabolites associated with Figure 4B The black point in each ROC curve presents the cutoff value with specificity and sensitivity.

To elaborate on the potential role of metabolites regulating the related pathway, we analyzed the dynamic change of abundance in five metabolites significantly associated with the above-mentioned metabolic pathways. Compared with the r3 group, 11-beta-hydroxyandrosterone-3-glucuronide (*p* = 0.023), 5-methoxyindoleacetate (5-MIAA, *p* = 0.030), 9,10-epoxyoctadecenoic acid (9 (10)-EpOME, *p* = 0.021), myo-Inositol (*p* = 0.020) and LysoPC (20:5) (*p* = 0.028) were significantly upregulated, while O-Phospho-4-hydroxy-L-threonine (*p* = 0.043) was significantly downregulated in the R3 group (Figure 5A). It was reported that 5-MIAA and 9 (10)-EpOME were closely linked with oxidative stress and environment changes (Li et al., 1996; Ho et al., 2001; Meatherall and Sharma, 2003; Newman et al., 2005; Gouveia-Figueira et al., 2018). However, rest of the metabolites including 11-beta-hydroxyandrosterone-3-glucuronide, myo-Inositol and PysoPC (20:5) involved with mechanisms in high-altitude area were rarely reported. To further assess the predictive potential of these six metabolites, the ROC curves showed significant AUC values for 11-beta-hydroxyandrosterone-3-glucuronide (AUC = 0.812, *p* = 0.019), 5-MIAA (AUC = 0.797, *p* = 0.025), 9 (10)-EpOME (AUC = 0.766, *p* = 0.041), and PysoPC (20:5) (AUC = 0.766, *p* = 0.041) (Figure 5B). Furthermore, the integration of these six metabolites using a generalized linear model resulted in an AUC value of 1 (*p* < 0.001, Supplementary Figure S5), demonstrating the outstanding discriminatory power of the multiple simultaneous metabolite classifier for HADAS.

## 4 Discussion

When descending to low altitude, it was a rigorous challenge for people who had adapted to the high altitude to lose hypoxia tolerance and physiological adjustments to re-acclimatize the hyperoxic environment. This phenomenon, also known as HADA, causes a series of clinical symptoms referred to as HADAS, involving cardiovascular, respiratory, neurological, and other systems. HADAS is also associated with reduced quality of life and can even be life-threatening because of severe HADAS-related complications such as thrombus and coagulation disorders (Zhang et al., 2012; Zhou et al., 2012; Zhou SY et al., 2012). The burden of HADAS highlights the need to improve the understanding for increasing the accuracy of early diagnosis, prompting intervention to prevent life-threatening complications.

Current studies reported that HADAS was associated with a high incidence of 71%–100% and had received increasing attention in recent years. However, its current diagnostic criteria, which are mainly based on HADAS scores according to the severity of symptoms (He et al., 2019), are mostly subjective and lack objective standards. Therefore, a crucial question that remains unanswered by previous works regarding individuals who returned to plain areas from a high altitude: is there an objective examination index that makes the diagnostic criteria of HADAS timelier and more precise? Thus, the present study compared the clinical characteristics of subjects exposed to a high altitude and returned to plain areas to explore such prospective and objective indices.

We compared different population groups under four physiological conditions according to different environments to analyze the variation of clinical examination indices. The findings revealed that the examination indices in our cross-sectional study changed significantly after moving to a high-altitude environment and normalized after living or returning to a low-altitude environment. The pathological changes observed in our samples were consistent with a recent longitudinal cohort study (Gao et al., 2023). We further analyzed the difference between subjects who returned from a high altitude to a plain area with almost no reaction and mild-to-moderate reaction according to HADAS symptom scores. There was no significant difference between the two groups of clinical data, indicating that the commonly used clinical examination indices were not specific in diagnosing HADAS. This prompted us to explore the disturbed mechanism underlying the difference in symptoms using a novel approach.

With reference to mechanisms of HADAS, myocardial zymography was previously reported as an important parameter in the evaluation of subjects with HADAS. Myocardial zymography, including creatinine kinase (CK), CK-MB, and lactate dehydrogenase, were assessed in HADAS individuals and their values on the third day were significantly lower than those values recorded at high altitude but still higher than normal (He et al., 2016; He et al., 2019), which was considered to be caused by H/R. Early studies have shown that H/R induced oxidative stress and production of ROS (Kim et al., 1998; Ozaki et al., 2003; Sureda et al., 2004). Meanwhile, serum superoxide dismutase and malondialdehyde levels were decreased when individuals returned to a lower altitude upon short-term exposure to a high altitude (Zhou et al., 2012). These findings informed us that HADAS might be associated with oxidative stress.

Metabolomics is the comprehensive analysis of metabolites, which are the end product of gene expression (Lawler et al., 2019). Metabolomics was used in this study, providing a reliable technique to expand the current understanding of the physiological process associated with HADAS. Although previous studies had reported the changes in metabolic levels under high altitude exposure, only one longitudinal cohort study used metabolomics to analyze human plasma samples following a return to seal level after being exposed to high altitude (Gao et al., 2023). That study found that high altitude exposure altered the metabolic characteristics, and after returning to a low altitude, a small portion of metabolites were still affected (Gao et al., 2023). However, while this study mainly focused on the long-term impact of exposure to high altitude on metabolism, it also shed light on the classification of metabolites through K-mean clustering. In the present study, we screened 235 metabolites that showed significant changes between subjects at plain and high altitudes, were also affected in subjects with HADAS and mild-to-moderate symptoms and returned to baseline in subjects with on reactions. Eleven targeted candidate metabolites, including 6-dehydrotestosterone-17-glucosiduronic acid, stearidonic acid, DGLA, dihydrouracil, dUMP, oleic acid, taurine, thromboxane A2, and three phosphatidylcholines were further verified. These findings supported previous literature and provided new insights into the mechanisms of HADAS.

Most importantly, to elaborate on the mechanisms underlying HADAS, we analyzed the difference among subjects affected by HADAS with mild-to-moderate and no-reaction symptoms. There were 193 significantly changed metabolites obtained in plasma samples. Meanwhile, we screened six metabolites that were significantly changed under linoleic acid metabolism, ascorbate and aldarate metabolism, glycerophospholipid metabolism, tryptophan metabolism, and vitamin B6 metabolism. These pathways were demonstrated to be among the top five enriched metabolic pathways potentially regulating the pathogenesis and development of HADAS. Specifically, linoleic acid metabolism and tryptophan metabolism have been reported to regulate the mechanisms of certain metabolites reported in previous studies, perhaps associated with the clinical symptoms of HADAS. Among these six metabolites, four were further screened and identified to be potential diagnostic indices of HADAS, including 11-beta-hydroxyandrosterone-3-glucuronide, 5-MIAA, and 9 (10)-EpOME, and PysoPC (20:5).

Previous studies reported the metabolism of tryptophan involves the 5-hydroxytryptamine, indole, and kynurenine pathways and plays an important role in protein biosynthesis. Multiple bioactive compounds produced by tryptophan metabolism could regulate various pathophysiological processes, including neuronal function, metabolism, oxidative stress, immune responses, and inflammatory responses (Xue et al., 2023). Gao et al. conducted a longitudinal cohort study using metabolomics and indicated that tryptophan metabolism was the most enriched metabolic pathway under hypoxia exposure at high altitude, suggesting that high-altitude exposure had a long-term impact on metabolism (Gao et al., 2023). In addition, our present study indicated that 5-MIAA in this pathway was significantly upregulated. However, there are only limited reports on the mechanism of action of 5-MIAA. 5-MIAA, formed through oxidative deamination, is the downstream metabolite of 5-Hydroxyindoleacetic acid in the 5-hydroxytryptamine pathway and belongs to the class of organic compounds known as indole-3-acetic acid derivatives (Meatherall and Sharma, 2003). Before 1996, 5-MIAA had been demonstrated to be present and synthesized in the pineal gland and organs such as the retina (Li et al., 1996). Similar to melatonin, Ho et al. revealed pineal 5-MIAA used light as an environmental cue, which could be another photoperiodic signal in animals (Ho et al., 2001). This feature of 5-MIAA might be associated with the neurological symptoms of HADAS, such as fatigue, sleepiness, and insomnia. Furthermore, 5-MIAA, discovered by non-targeted metabolomics in intestinal contents and liver, was demonstrated to activate the Nrf2 pathway to improve oxidative stress response *in vitro* (Luo et al., 2022).

Another metabolite identified to change significantly after returning to plain areas from high altitudes worth highlighting was 9 (10)-EpOME that upregulated in the linoleic acid (LA) metabolic pathway. LA, mostly derived from vegetable oils, nuts, meats, and eggs in human diets, is the most abundant polyunsaturated fatty acid, which regulates the homeostasis of inflammation, Vaso tension, and other physiologic processes. Generally, LA is converted into 9 (10)-EpOME and 12 (13)-EpOME through a cytochrome P450 (CYP)-dependent metabolism and then metabolized by soluble epoxide hydrolase to 9,10-dihydroxyoctadecenoic acid (9,10-DiHOME) and 12,13-dihydroxyoctadecenoic acid (12,13-DiHOME) (Newman et al., 2005). Previous studies demonstrated that 9 (10)-EpOME and 9 (10)-DiHOME, which are associated with pulmonary toxicity, could be produced by neutrophils in the lung after exposure to hyperoxia, suggesting that these metabolites might be part of the inflammatory response to environmental insults (Newman et al., 2005; Gouveia-Figueira et al., 2018). Moran et al. compared the relative toxicity and mechanisms of LA, EpOMEs, and DiHOMEs in the renal proximal tubule of rabbits and revealed that the toxicity of LA and EpOMEs induced the uncoupling of oxidative phosphorylation (Moran et al., 2000). The underlying mechanism of 9 (10)-DiHOME toxicity was regarded as disrupting mitochondrial function (Sisemore et al., 2001). Another component of that toxic response to 9 (10)-EpOME was its ability to disrupt cardiovascular function (Siegfried et al., 1990). Henning et al. reported that LA-induced endothelial cell dysfunction in atherosclerosis was mediated by oxidative stress (Hennig et al., 2001). Besides the high concentrations of LA, 9 (10)-EpOME could induce oxidative stress and activate NF-κB and AP-1 transcription factors (Viswanathan et al., 2003). However, recent studies on cardiovascular toxicity were focused on 12 (13)-EpOME. Hence, the detailed mechanisms of 9 (10)-EpOME in cardiovascular function were still less clear. In summary, the present study provides a promising approach to exploring the mechanisms of HADAS.

Nonetheless, there are three major limitations in this study should be addressed in future research. Firstly, the sample size in each group may not be sufficiently robust for more convincing statistical measurements. Secondly, given that the study involves four groups from varied environments and limited access to certain populations, factors influencing human metabolism, such as diet and exercise habits, should be better standardized or incorporated as parameters in subsequent investigations. Thirdly, the presence of certain isomers with similar retention times and structures poses challenges in distinguishing each isomer solely through mass spectrometry or chromatography-mass spectrometry. Despite these limitations, our results remain reliable and informative, providing enhanced insights into HADAS. Further research should delve into the detailed mechanistic processes of the identified key metabolites.

## 5 Conclusion

This cross-sectional study assessed the human plasma samples with HADA after exposure to high altitudes and returning to plain areas. The levels of four metabolites demonstrated significant differences between the r3 and R3 groups. These metabolites may be involved in the pathogenesis of HADAS via relative pathways mediated through oxidative stress and could improve the understanding of HADA to explore prospective diagnostic indices. In addition, metabolomics, which played a more efficient role than clinical examination in detecting the changes in metabolism underlying the difference in symptoms, provided new insights into molecular mechanisms of HADA.

## Data Availability

The original contributions presented in the study are included in the article/Supplementary Material, further inquiries can be directed to the corresponding authors.
