# Supplementary figures and images for: Assessment of metabolomic variations among individuals returning to plain areas after exposure to high altitudes: a metabolomic analysis of human plasma samples with high-altitude de-acclimatization syndrome
Source: Front Mol Biosci. 2024 Jun 19;11:1375360. doi: 10.3389/fmolb.2024.1375360 (PMC11220191; doi:10.3389/fmolb.2024.1375360)

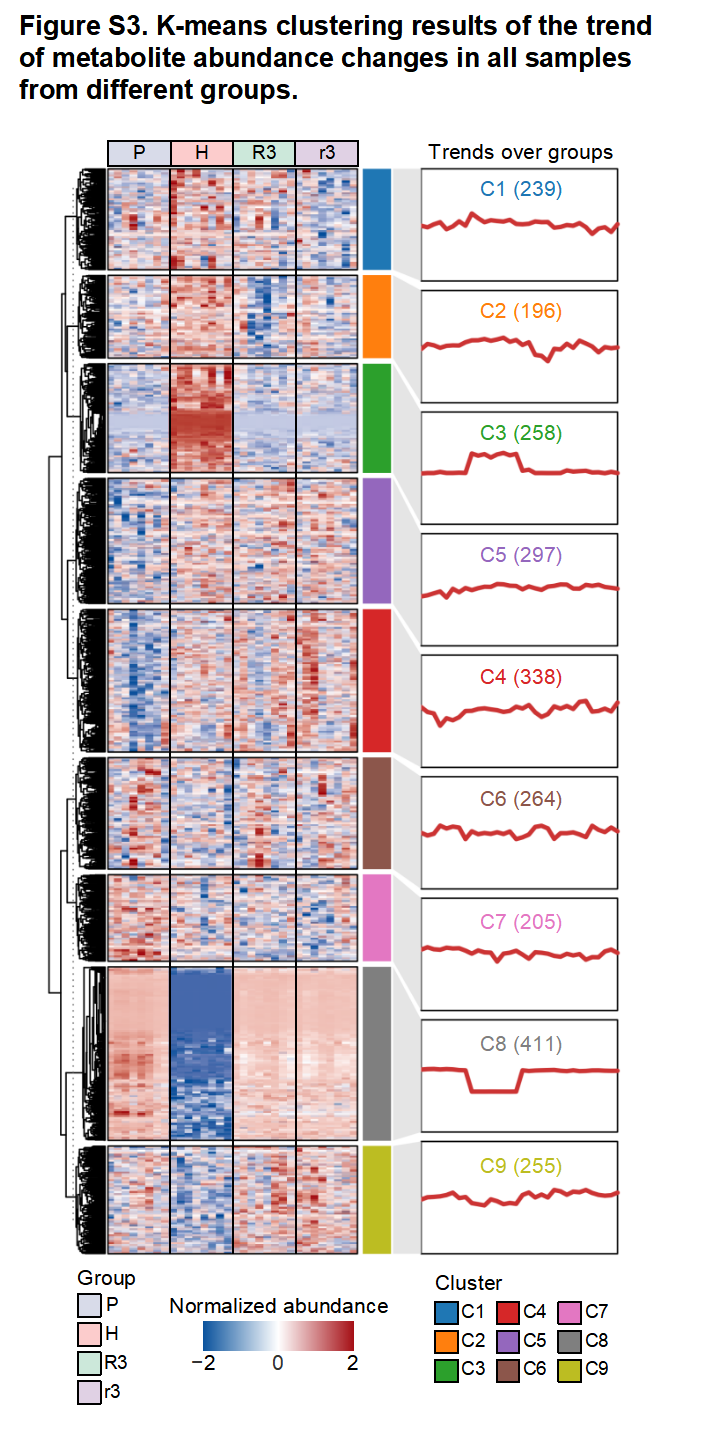

Supplement: Supplementary file 1 [file Image3.TIF]

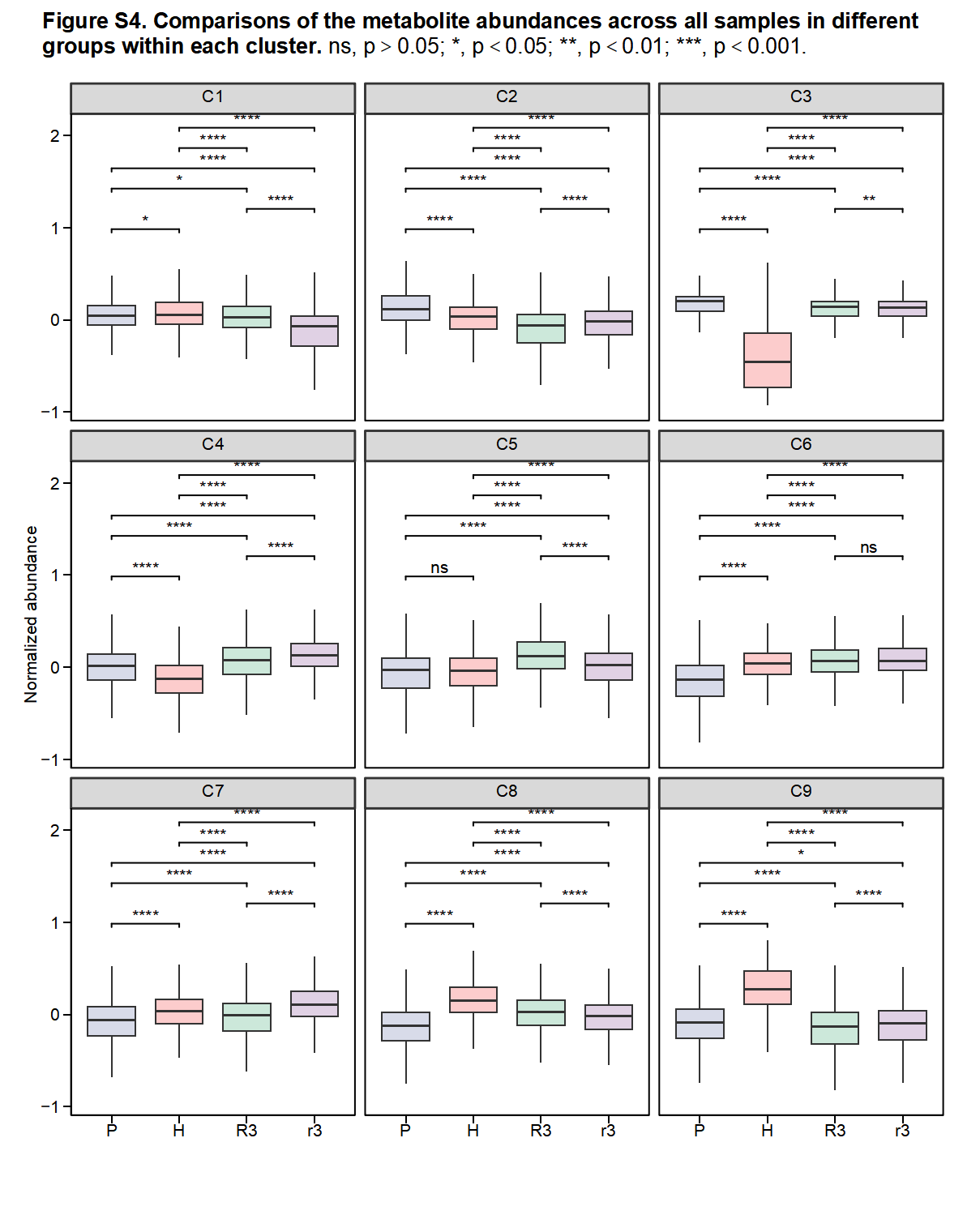

Supplement: Supplementary file 2 [file Image4.TIF]

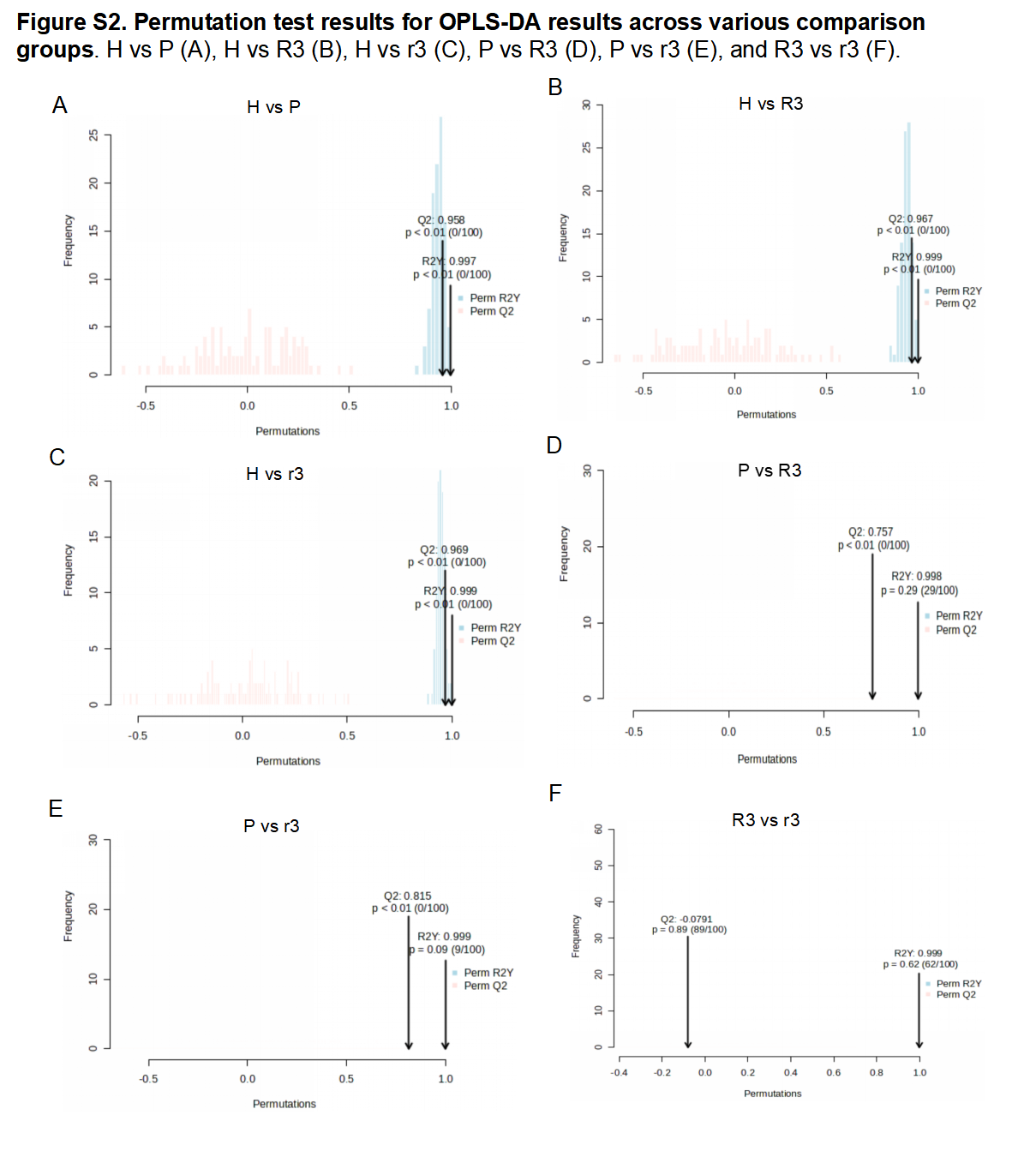

Supplement: Supplementary file 3 [file Image2.TIF]

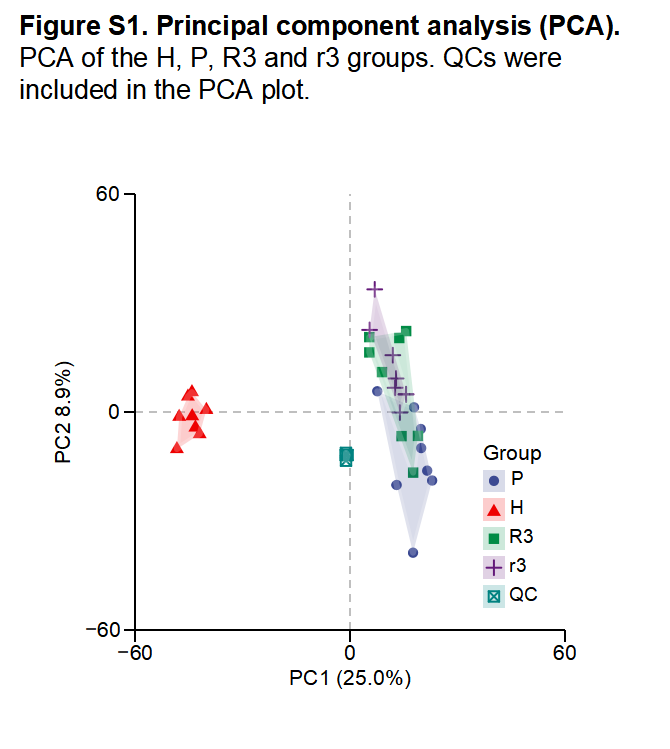

Supplement: Supplementary file 4 [file Image1.TIF]

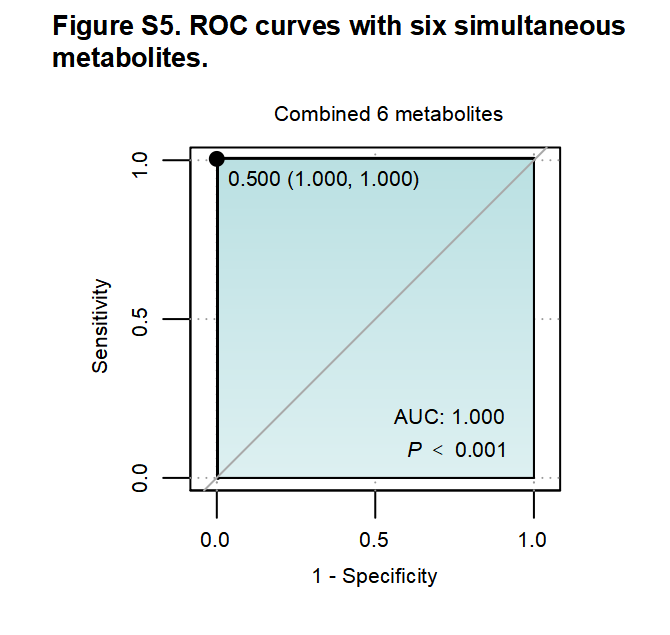

Supplement: Supplementary file 6 [file Image5.TIF]
